# Council on Defense of Medical Research of the American Medical Association Invokes the Aid of Medical Journals in Its Efforts to Prevent or Overcome Hostile Public Opinion—Letter of Dr. W. B. Cannon, Chairman

**Published:** 1909-01

**Authors:** Walter B. Cannon

**Affiliations:** Chairman. Boston


**Council on Defense of Medical Research of the American Medical Association Invokes the aid of medical journals in its efforts to prevent or overcome hostile public opinion—Letter of Dr. W. B. Cannon, Chairman.**

*Editor Buffalo Medical Journal:*

SIR:—The Council on Defense of Medical Research of the American Medical Association is desirous of obviating, as completely as possible, any cause for complaint against animal experimentation. Much of the "evidence" cited by hostile agitators is taken from articles in journals devoted to the medical sciences. Instances are frequently cited in which it is stated that, as there is no mention of anesthetics, the work must have been done without anesthesia.

Will you not aid the Council in its efforts by very careful examination of articles submitted to you for publication, with especial reference to the use of words likely to cause misapprehension regarding the experience of the animals used for research? And in every instance in which anesthesia is a condition in the investigation, will you not point out to authors the importance of making that fact prominent? We hope that by the coöperation of all who are interested in the promotion of medical science, the development of a public opinion hostile to medical research may be checked and that there may be a growth of popular understanding of the aims, the methods, and the significance of the results of animal experimentation.

Yours sincerely,
WALTER B. CANNON,
Chairman.

Boston, December 1, 1908.

